# Biogenesis, Isolation, and Detection of Exosomes and Their Potential in Therapeutics and Diagnostics

**DOI:** 10.3390/bios13080802

**Published:** 2023-08-10

**Authors:** Smrity Sonbhadra, Mehak, Lalit M. Pandey

**Affiliations:** Bio-Interface & Environmental Engineering Lab, Department of Biosciences and Bioengineering, Indian Institute of Technology Guwahati, Assam 781039, India; s.smrity@iitg.ac.in (S.S.); mehak1998@iitg.ac.in (M.)

**Keywords:** exosomes, biomarkers, micro-RNA, biosensors, diagnostic applications, therapeutic potential

## Abstract

The increasing research and rapid developments in the field of exosomes provide insights into their role and significance in human health. Exosomes derived from various sources, such as mesenchymal stem cells, cardiac cells, and tumor cells, to name a few, can be potential therapeutic agents for the treatment of diseases and could also serve as biomarkers for the early detection of diseases. Cellular components of exosomes, several proteins, lipids, and miRNAs hold promise as novel biomarkers for the detection of various diseases. The structure of exosomes enables them as drug delivery vehicles. Since exosomes exhibit potential therapeutic applications, their efficient isolation from complex biological/clinical samples and precise real-time analysis becomes significant. With the advent of microfluidics, nano-biosensors are being designed to capture exosomes efficiently and rapidly. Herein, we have summarized the history, biogenesis, characteristics, functions, and applications of exosomes, along with the isolation, detection, and quantification techniques. The implications of surface modifications to enhance specificity have been outlined. The review also sheds light on the engineered nanoplatforms being developed for exosome detection and capture.

## 1. Introduction

Exosomes are nano-sized extracellular vesicles having a diameter in the range of 30–150 nm [1]. They are found to be secreted in various body fluids. Exosomes possess lipid bilayer membrane structure and a variety of cellular components that are biologically active. Exosomal nucleic acids and proteins play an important role in intercellular communication or information exchange and signal transduction [2]. Isolation, detection, and quantifications of exosomes are essential for their potential applications. There are numerous methods available for the isolation and detection of exosomes. Ultracentrifugation (considered as the gold standard), precipitation, immunoaffinity, and size exclusion chromatography are some of the conventional methods, whereas microfluidic-based separation techniques, including nano-biosensors, lab-on-chips, and nanoplatforms are being recently studied to develop next-generation and highly efficient separation methods [3].

Nanomaterials or nanocomposites are of great interest, owing to their large surface area-to-volume ratio, which can help in increasing exosome capture efficiency and magnetic properties (especially for magnetic nanoparticles) for easy separation. In a study, Fang et al. [4] summarized the use of nanomaterials for exosome isolation and analysis for the detection of diseases via liquid biopsy. Nanomaterials also act as signal transducers and signal amplifiers for the molecular detection of exosomes. Moreover, surface modification of nanomaterials using organic and inorganic moieties and conjugation to aptamers or target-specific antibodies can be carried out to enhance exosome capture. Once the exosomal bodies are isolated and detected, they further need to be characterized and quantified on the basis of their size, number, concentrations, and purity. Some of the commonly used techniques for this purpose involve nanoparticle tracking analysis (NTA) [5], dynamic light scattering (DLS) [6], tunable resistive pulse sensing (TRPS) [7], Western blot [8], atomic force microscopy (AFM), spectrophotometry [9], and transmission electron microscopy (TEM) [10].

There is growing research in this field, as exosomes can serve as potential diagnostic biomarkers for the detection of several diseases, for example, neurodegenerative diseases, cancer, autoimmune disorders, and diabetes [11]. This could be attributed to the various biomarkers, CD63, CD81, CD151, CD9, GPC, and several proteins, HSP70 and HSP90, expressed on the surface of exosomes [12]. Exosomes could also act as therapeutic agents against several diseases, for instance, liver disorders, kidney dysfunctions, skin burns, cancer, central nervous system diseases, and diabetes and as therapeutic cargo delivery agents [13,14]. There are various studies suggesting that exosome-derived miRNAs, such as miR-587, miR-298, miR-4443, miR-450-2-3p, miR-21, miR-4454, miR-125b miR-223, miR-29, miR-103, miR-107, and let-7, could act as both diagnostic markers and therapeutic agents [15,16]. One such study was performed by Giau et al. [17], who extensively reviewed the advances in research of exosomes and exosomal miRNAs as biomarkers for disorders like Alzheimer’s disease.

The available literature on exosomes provides brief insights on their biogenesis, detection and isolation techniques, and therapeutic potential, with a little focus on the technological advancements for the detection and isolation of exosomes [18,19,20]. Herein, we will address the biological as well technological aspects in the field of exosomes. The advent of nanobiotechnological approaches is highly crucial for designing nanoplatforms and nanobiosensors for the efficient isolation and detection of exosomes. These advancements have led to the design of efficient lab-on-chip nanobiosensors possessing a low limit of detection and multifunctionality. Further, surface modifications impart the desired specificity.

**Figure 1:**
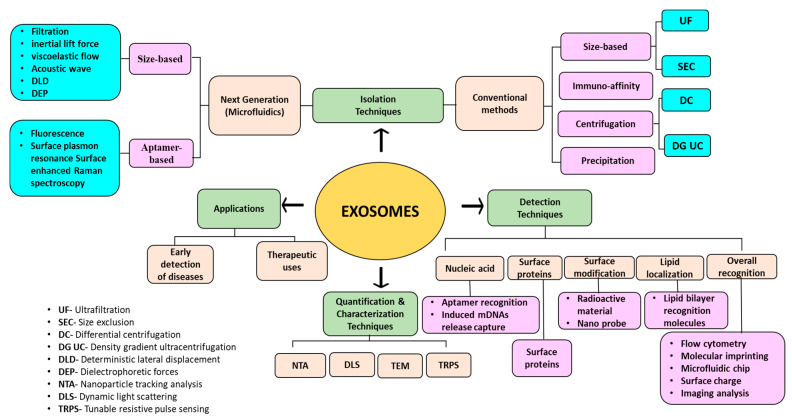
Graphical abstract portraying the techniques involved in the isolation, detection, quantification, and characterization of exosomes.

In this regard, this review will briefly summarize the history and biogenesis of exosomes and their characteristics. Traditional as well as emerging methods of isolation and detection along with the quantification and characterization techniques of exosomes will be discussed. The recent developments in fabrication and surface modification approaches of microfluidic-based nano-devices for the isolation and detection of exosomes will also be outlined. This review will also shed light on various functions and applications of exosomes. The studies on technological development and advancement in the field of nano-biosensors and nanocomposites for the early detection of diseases will be highlighted along with the therapeutic potential of mesenchymal and cardiac cell-derived exosomes against several diseases, as shown in Figure 1. The current status of using exosomes in clinics as therapeutic agents against various diseases will also be discussed. Challenges and future prospects of the use of exosomes for clinical purposes will be highlighted. Two comprehensive tables will be provided for the isolation and diagnostic potentials of exosomes. Another table will list the clinical status of exosome-based therapeutics.

## 2. Exosomes

### 2.1. History and Biogenesis of Exosomes

In the beginning, when the extracellular vesicles (EVs) were first discovered, these micro-vesicles along with the exosomes were seen as cellular wastes or the by-products/debris of cells [21,22]. The exosomes were first discovered in the year 1983, as described in two different research works by Harding et al. and Pan et al. The movement of transferrin receptors (TfRs) was observed onto the maturing reticulocytes from the plasma membrane. These TfRs were found to be taken up by the cells, further reassembling in the form of tiny vesicles within them [23]. These small vesicles were later given the name “exosomes”, seen as a product secreted from the maturing red blood cells, thereby discarding the initially assumed fact that the exosomal vesicles are waste, destined to be destroyed by the trafficked lysosomes [24].

EVs are found to be secreted from almost all types of cells and are majorly classified as exosomes, microvesicles (MVs), and apoptotic bodies depending upon their structure, size, and origin of formation [25]. Various EVs fuse to produce an endosome, which further develops into multivesicular bodies (MVBs). During the formation of MVBs, intraluminal vesicles (ILVs) carrying the cytosolic content are generated by the invagination of the outer membrane of the endosomes [26]. The diameter of ILVs varies in the range of 50 to 90 nm. The synthesized MVBs are of two main providences, viz., intermediate formation involved in intracellular protein degradation or exosome synthesis [27]. Though the factors determining the fate of an MVB are not very clearly understood, some studies reveal that the destiny of a particular MVB depends on the amount of cholesterol present. So, the one with a higher level of cholesterol is preferably selected for further secretions, and the rest (with a low cholesterol concentration) are sent for lysosomal degradation [28,29]. Now, unlike the generation of other MVBs, which are mainly formed from “budding off” of the cell membrane, the biosynthesis of exosomes involves much more complexity. It includes the reverse membrane enfolding and dispensation of the MVBs, leading to the release of exosomes into intercellular fluid [30]. So, the exosomes are believed to be secreted when the secretion of plasma membrane-fused MVBs takes place [31,32,33]. Furthermore, the cargo of exosomes represents its fidelity by demonstrating the molecular processing part, taking place within the parent cell, and serves as a potential surrogate of the respective parent cell in the body fluids [30].

**Figure 2:**
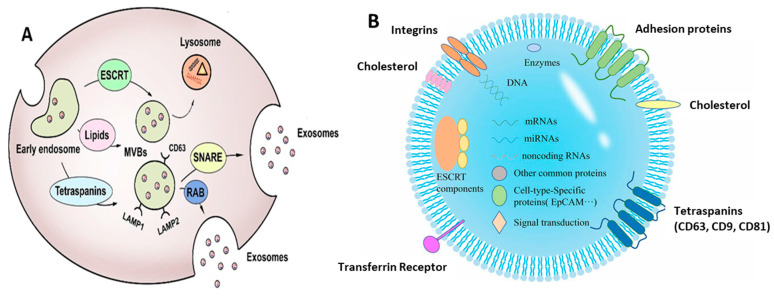
(**A**) Biogenesis of exosomes showing their origin and release. Adapted from Liu et al., 2021 [46]. (**B**) Schematic representation of exosomal composition with its significant biomarkers. Adapted from Chen et al., 2022 [47].

Moreover, the MVBs, along with the casing of their cargo protein, turn to the endosomal sorting complex required for transport (ESCRT), like the ESCRT-0, 1, 2, 3 associated with some proteins, for example, Vps4, Flotillin, Tsg101, and Alix. Formation of the budding membrane takes place when the ESCRT-0 complex associates with ESCRT complexes 1 and 2 after the membrane deformation. Altogether, these complexes meld with ESCRT-3 and Vps4 protein, giving rise to the formation of ILVs by cleaving the neck of the buds formed [7,34]. Figure 2A depicts the biogenesis of exosomes. However, there exist some ESCRT-independent pathways as well for exosome formation, using raft-associated lipid microdomains (present on the cell membrane) and certain linked proteins (like the tetraspanins). The sphingomyelinases-augmented lipid rafts convert sphingomyelin to ceramide, which induces a spontaneous budding of the membrane, forming ILVs [35,36]. The exact contribution of tetraspanin proteins in vesicle formation is not yet completely understood. However, the tetraspanin-enriched microdomains (TEMs) are associated with the processes like loading of cargo molecules, grouping particularly targeted receptors, and processing the essential molecular components into exosomes [37,38].

Exosomes act as a very crucial source of cellular dumping, enclosing within them various biomolecules, like proteins, lipids, and nucleic acids (DNA, mRNA, miRNA, etc.) [39,40,41]. Figure 2B depicts the b composition of exosomes, along with the biomarkers expressed on their surface. Thus, the exosomes play a vital role in multiple physiological and pathological states of the body, serving as blueprints of their parental cells [42,43,44,45]. In fact, exosomes are associated with a plethora of functions and applications, as discussed in the following sections.

### 2.2. Key Characteristics

Exosomes are known to be of endosomal origin. These are tiny (virus-sized) vesicles, appearing as flat spherical structures [48,49]. Exosomes are membranous vesicles spanning in the diameter range of 30–150 nm [28,50]. Being derived from MVBs, exosomes possess a lipid bilayer that is rich in unsaturated fatty acids, phosphatidylserine, polyglycerin, sphingomyelin, cholesterol, and gangliosides. The density of exosomes varies in the range of 1.08 to 1.22 g/mL [51,52]. The lipid bilayer of the exosomes is specifically known to provide them with strength, firmness, and an armored internal environment acting as a barrier, thwarting their enzymatic degradation and providing them higher stability as a carrier molecule [25]. Comparing the lipid profile of the exosome’s membrane with their parent cells, there appear to be some minor differences, as the newly synthesized ones carry elevated levels of lipids, like cholesterol, phosphatidylserine, and sphingomyelin and a fewer lyso(bis)phosphatidic acids and phosphatidylcholine [53].

Exosomes are originated from the endocytic compartment of the producer cell and are secreted from almost all the types of cells in the body fluid, including saliva, sputum, tear, urine, bile, blood, bronchoalveolar lavage fluid, semen, alveolar fluid, pleural fluid, bronchial fluid, cerebrospinal fluid, vitreous, ascites, and breast milk [54,55,56]. Being endosomal in origin, these are explicitly embellished with the endosomal components as “specific biomarkers” (CD63, CD9, and CD81), diverse proteins like the transcription factors, and oncogenic regulators. The protein constituents of EVs act as a fine indicator reflecting the subtype of EV, giving information about the course of biogenesis, the way of release, and the original cell form [57]. There exist some common proteins existing in all forms of exosomes (regardless of the cell type they originate from), including heat shock protein (HSP84, HSC70, and HSP90β), tumor susceptibility gene 101, and Alix [58]. Exosomes also carry genes from their parental cells like microRNAs, long non-coding RNAs, and circular RNAs [59,60].

## 3. Isolation Methods for Exosomes

The first thing to be done for exploring exosomes is to find an efficient and productive way of extracting and purifying exosomes, as they carry an immense potential to be used as diagnostic markers for various diseases and as drug therapy carriers. Bearing diversified size ranges and varying contents of protein and nucleic acid, exosomes can be extracted from multiple sources of cell cultures or body fluids using different methods of separation [61]. But due to the smaller size of exosomes and lesser availability in live samples, their isolation and purification process become a considerable challenge. The following sections illustrate various isolation techniques used for the isolation of exosomes. 

### 3.1. Ultracentrifugation

**Figure 3:**
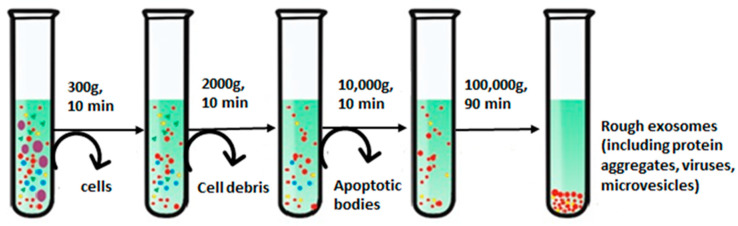
Step-wise isolation of exosomes using differential ultracentrifugation. Adapted from Yang et al., 2020 [64].

The basic principle underlying the ultracentrifugation (UC) step is owing to the diverse sedimentation coefficients of the particles present in a sample, which leads them to settle down in different layers that can be collected separately [62]. For instance, exosomes form a distinct fraction of the zone, floating on the top of a sucrose gradient and attaining an equilibrium density (1.10 to 1.21 g/mL), providing their easy recovery [48]. UC can be classified as differential centrifugation and density gradient centrifugation. Differential centrifugation is performed at gradually rising speeds, which has been most widely used for the isolation of exosomes [63], as shown in Figure 3 [64]. Bigger cells and their wastes are separated at 300 to 2000× *g*; further increasing spin speed to 10,000× *g* removes bigger EV molecules and remaining cellular by-products, and ultimately, ultracentrifugation at a spin speed of 100,000× *g* segregates exosomes [65].

Furthermore, the purity of the process is affected by the centrifugation time and using sucrose as density gradient centrifugation. Density gradient centrifugation distinguishes samples as per their densities, with exosomes lying in a 30% sucrose base material. This isolates them from other non-exosomal substances, which could otherwise be precipitated during ultracentrifugation [66]. Various studies have reported the use of iodixanol over the sucrose method for performing density gradient centrifugation. Iodixanol enables the production of an isosmotic solution with varying densities to preserve the shape and size of vesicles while moving along the gradient [67]. The iodixanol-based approach also overcomes the limitations procured with sucrose as a gradient substance due to its large viscosity and hyperosmotic properties, harming the exosomes and taking longer durations to be sedimented [68,69]. Generally, an extra UC step is also carried out in phosphate-buffered saline for washing exosome pellets and reducing the chances of protein contamination for better purity [70]. The UC method is known to be of high throughput, being widely used these days for exosome extraction, and hence allowing it to be a “gold standard” for exosome isolation [62]. Though the method possesses several advantages over others, it also involves a few limitations. One of the common issues is related to the high viscosity of biofluids, which requires multiple UC steps for longer durations, thereby compromising the integrity of exosomes [33,71].

### 3.2. Precipitation

**Figure 4:**
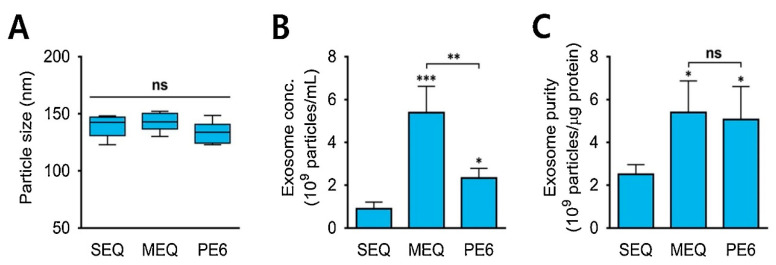
(**A**) The average size of exosomes isolated from urine samples comparing three different modes of precipitation strategies. (**B**) Concentrations of isolated exosomes from urine (exosomes/mL). (**C**) Purity of the isolated exosome particles (exosome particles/µg protein from the urine sample). * *p* <  0.05, ** *p*  <  0.01, *** *p*  <  0.001, ns: no significance; n  =  3. Adapted with permission from Cho et al., 2020 [76]. Copyright 2020 Elsevier.

In the precipitation method, the solubility of exosomes is altered by adding them into a solvent, which results in their precipitation out of the solution. Usually, water-excluding polymers are used that cause aggregation of water droplets with each other and force the insoluble molecules from water to pop out of the solution [72,73]. Precipitation with polyethylene glycol (PEG), having a molecular weight of 8 kDa, is commonly used for this purpose. The PEG surrounds the exosomes, causing their aggregation, followed by precipitation of exosomes under low-speed centrifugation [72]. Nowadays, commercially available isolation kits, like Mini and RNeasy MinElute Cleanup kits (Qiagen; Valencia, CA, USA), ExoQuick ULTRA isolation kit for Serum and Plasma (Cat #EQULTRA-20A-1) (System Biosciences, Palo Alto, CA, USA), and MagCapture™ Exosome Isolation Kit PS Ver.2 (FUJIFILM Wako Pure Chemical Corporation, Chuo-ku, Osaka 540-8675, Japan), are also widely used to separate exosomes from other biological samples (protein, miRNA, and mRNA) via the precipitation method [74,75]. Figure 4A shows the average diameter of exosomes (120–150 nm) isolated from urine samples using the precipitation approach. Standard ExoQuick-TC™ (SEQ), modified ExoQuick-TC™ (MEQ), and PEG6000 (PE6)-based precipitations were used in the study [76]. Figure 4B,C depict the concentrations and purity of isolated exosomes, respectively. The concentrations of isolated exosomes varied in the range of 0.8 to 5.4 × 10^9^ particles/mL [76]. MEQ resulted in the highest concentration and better purity, highlighting the role of polymers in this method.

Using the precipitation method, the mass separation of exosomes becomes possible without affecting their biological activities. PEG-based precipitation was used by Weng et al. to isolate exosomes from the floatable component of the cell culture, and the morphology of exosomes was clearly observed using an electron microscope [77]. The advantages of the PEG-based isolation method include simplicity in operation, high efficiency, and low cost. Talking about the drawbacks, the exosomes isolated using this method might carry a certain amount of impurities, such as polymers. Protein-organic solvent precipitation (PROSPR) is one of the other efficiently used precipitation-based methods to isolate exosomes using organic solvents (acetone, trichloroacetic acid, and chloroform). The organic solvents cause precipitation of the soluble proteins, leaving behind exosomes in the supernatants, which are then concentrated by vacuum concentrators or filtration methods [78].

### 3.3. Immunoaffinity-Based Capture (IAC)

**Figure 5:**
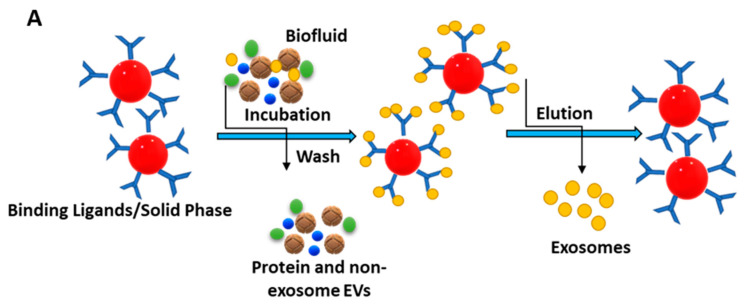

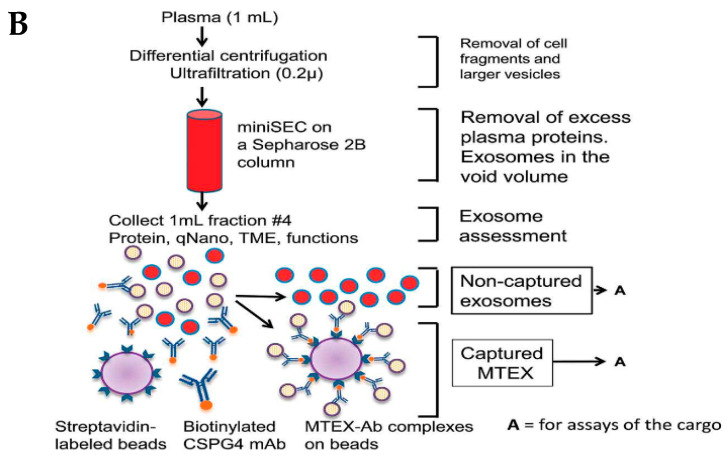
(**A**) Principle behind immunoaffinity-based method for isolating exosomes. Adapted and recreated from Yang et al., 2020 [64]. (**B**) Schematic diagram showing step-wise isolation of exosomes from MTEX by the capture of mAb 763.74 from bulk of non-aggregated exosomes. Adapted from Sharma et al., 2018 [82].

The immunoaffinity technique for isolating exosomes is attained via labelling specific membrane proteins, which are available in abundance, like CD9, CD63, CD81, ALIX, EPCAM, RAB5, and ANNEXIN [79]. The schematic illustration of the immunoaffinity-based isolation of exosomes is presented in Figure 5A.

The immune separation method can be further divided into three types depending on the variation in coated antibody substrates, such as chromatographic stationary phase separation, magnetic bead immune separation, and enzyme-linked immunosorbent separation. Enzyme-linked immunosorbent assay (ELISA) is one common method to isolate and quantify exosomes using IAC, with the help of biomarkers available on the surface [80]. These membrane peptides can acquire precise binding with similar antibodies on distinctive cargo substrates, thus aiding the isolation of exosomes [81]. This method is further being extended to use noncovalent interactions, allowing a more detailed dismantling of exosomes from the magnetic beads, rendering purer exosomes. In a study, melanoma cell-derived exosomes (MTEXs) were isolated using a monoclonal anti-chondroitin sulfate peptidoglycan (CSPG4) antibody 763.74 aided by streptavidin-attached magnetic beads, providing an efficiency of 98% exosome capture [82]. Figure 5B shows the capture and isolation of exosomes using the IAC approach with a monoclonal antibody specific to CSPG4 [82].

The separation efficacy of the IAC process is small, and the activity of exosomes is prone to the change in pH and salt concentration [83].

### 3.4. Size-Based Isolation Method

Based on differences in the particle size of analytes, exosomes are commonly known to be separated using two methods, i.e., ultrafiltration and size exclusion chromatography (SEC) [84].

#### 3.4.1. Ultrafiltration

For the ultrafiltration method, the separation efficacy of exosomes relies mainly on the size and molecular weight of the suspended particle in a sample [85]. Membrane filters with a pore size of 0.22 μm are usually utilized for isolating exosomes from the filtrate [86]. Before starting the filtration step, membrane filters having pore sizes of 0.80 μm are used to remove the cells, debris, and larger vesicles from retentate fluid containing exosomes [87]. The sample pre-treatment process is simple, and exosomes can be separated effectively from impurities (proteins and lipids), maintaining their probity and biological activity. But it requires a relatively superior quality of chromatographic column for separation, making it a costlier approach. The separated exosomes are prone to being damaged by the application of external pressure and are also prone to pore blocking, hampering the separation performance [88,89]. One of the other downsides of this technique involves its lower performance efficiency, as filter units are highly prone to clogging. To combat this issue, tangential flow filtration (TFF) or cross-flow approach is applied, where pressure is provided in a perpendicular direction, altering the hydrodynamic flow forces, unlike the normal or the dead-end ultrafiltration method (where flow is directed in the direction of pressure drop). In TFF, the pressure drop is engineered in such a way that there is a component of flow tangential to the filter rather than through it, allowing the system to run continuously and with less clogging. Thus, this enables the isolation of exosomes from active biological samples without leading to vesicle deformation or lysis [90,91].

**Figure 6:**
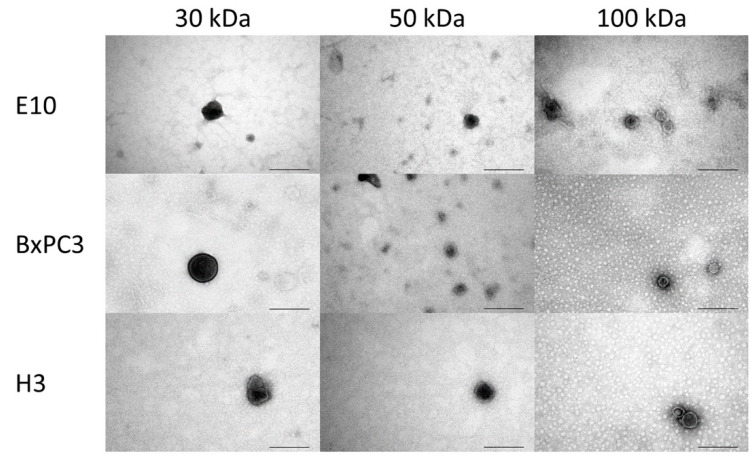
TEM images of exosomes isolated from three different cell lines (H3, BxPC3, and E10) using the ultrafiltration method, having molecular weight cut-off values of 30, 50, and 100 kDa. Scale bar: 200 nm. Adapted from Eduarda et al., 2018 [95].

Sometimes, these two types of ultrafiltration methods are combined, known as a hybrid approach, where the sample is first introduced along one side of a membrane, followed by injecting the sample from the opposite side of the membrane and further repeating the cycle for a higher recovery of exosomes and lower fouling [92]. Parimon et al. [93] used the ultrafiltration method for isolating exosomes from bronchoalveolar lavage fluid using a 100 kDa molecular weight nano-membrane filter. The system was proved to be efficient in isolating exosome particles with a limit of detection (LOD) value of 7.69 ± 2.6 × 10^8^/μL. In another study, an approach using ultrafiltration combined with sequential centrifugation was utilized for the isolation of exosomes from human colon cancer samples, which was found to be capable of performing impartial isolation of exosomes from the conditioned media having uniform vesicle size [94]. Figure 6 shows transmission electron microscopy (TEM) images of exosome particles isolated successfully from different cell lines, i.e., H3, BxPC3, and E10, using ultrafiltration with three molecular weight cut-off values of 30, 50, and 100 kDa. The exosomes were spherical with a diameter of 60–140 nm. Molecular weight cut-off values of the membrane did not show any significant impact on particle size and morphology [95].

**Figure 7:**
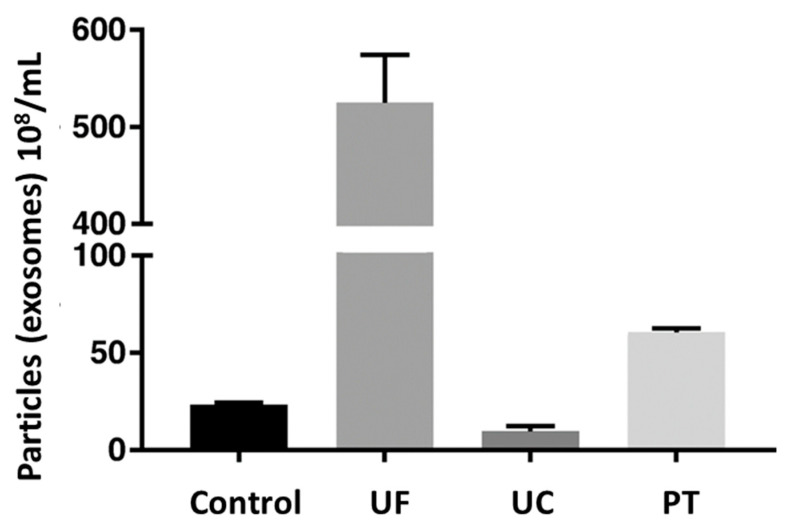
Comparative efficiency of different techniques by measuring exosomal concentration (10^8^/mL) of sample. Here, control is native cell culture supernatant; UF is ultrafiltration; UC is ultracentrifugation; and PT is precipitation. Adapted from Klymiuk et al., 2019 [96].

A comparative study of different techniques was performed to isolate exosomes (particles/mL) from equine mesenchymal stem cells, as shown in Figure 7. The exosome concentration in control, i.e., cell culture supernatant, was 2.32 × 10^9^ particles/mL. Among the three techniques, the highest mean concentration of exosomes was acquired for the ultrafiltration technique, i.e., 52.5 × 10^9^ exosomes/mL, followed by precipitation (6.06 × 10^9^ exosomes/mL), and the lowest for ultracentrifugation (9.6 × 10^8^ exosomes/mL) [96]. This indicated that ultrafiltration and precipitation concentrated the control sample, while ultracentrifugation resulted in a loss of exosomes.

#### 3.4.2. Size Exclusion Chromatography

As a mixture of molecules is passed through a column of polymeric beads having multiple pores, individual molecules pass down the pores depending on their size. Molecules like exosomes possessing larger hydrodynamic radii are not able to enter the small pores and pass through the column quicker, whereas molecules with a smaller radius get stuck within the pore and migrate via the channels of the column, eluting out later from the column [84]. In a study carried out by Hong et al. [97], exosomes were isolated from acute myeloid leukemia (AML) plasma using mini-sized SEC columns, having a packing of sepharose 2B. This method was found to be efficient in isolating clear and non-aggregated exosomes having a size range of 50 to 200 nm [97]. In another study, a single-step SEC was performed for isolating exosomes from body fluids using a sepharose CL-2B column. The method effectively separated the vesicles having a diameter of more than 75 nm [98].

**Figure 8:**
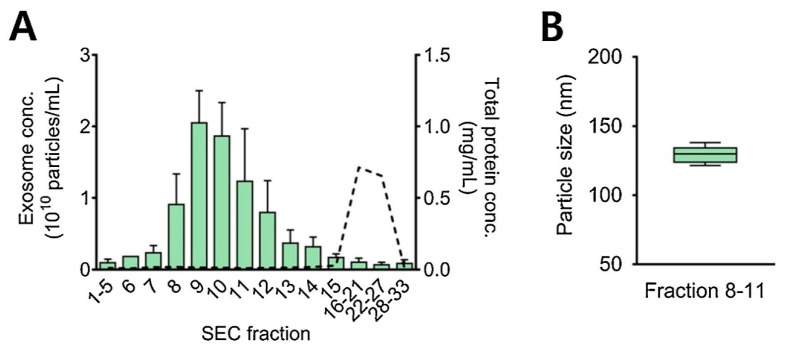
SEC—based isolation of exosomes from 1 mL of urine sample. (**A**) *Y*-axis shows exosome concentrations (particles/mL) and total protein concentrations in mg proteins/mL from all the collected SEC fractions. Bars represent exosome concentration and dotted line refers to protein concentration. (**B**) Average size of urinary exosomes isolated during SEC fractions 8–11. Adapted with permission from Cho et al., 2020 [76]. Copyright 2023 Elsevier.

It is also practical and wise to combine the two methods (ultrafiltration and size exclusion chromatography) for better separation, more efficient purification, and improved yield of exosomes [61]. Here, firstly ultracentrifugation is used to separate EV particles from the sample, followed by the SEC for the enrichment of exosome particles [99]. Figure 8 represents exosome concentrations (particles/mL) in different fractions. Proteins were eluted after the exosomes. The average concentration of isolated exosomes was found to be 8.1 × 10^9^ particles/mL in fractions 8 to 11 with a diameter of 120 to 150 nm [76].

### 3.5. Microfluidic Separation

A microfluidic separation system analyzes and manipulates small volumes of fluids, having a range from µL to mL using channels with micro-dimensions on a single chip. A microfluidic system enables immunocapture, characterization, as well as quantification of exosomes from a cell culture medium or a biological sample [100]. The different components of a microfluidic device include micromixers, valves, microchannels, and pumps [101]. The various approaches used within the microfluidic separation method have gained popularity lately for the separation and purification of exosomes bearing differences in their physical and biochemical properties [102]. Some of the common approaches used within the microfluidic devices include inertial lift force, viscoelastic flow, filtration, deterministic lateral displacement (DLD), acoustic waves, dielectrophoretic forces (DEPs), and immunoaffinity-based methods for the isolation of exosomes [103].

Wu et al. [104] used a mixture of acoustics and microfluidics, i.e., acoustofluidics, for isolating exosomes from whole blood including a microscale cell removal module (for separating bigger blood components) and an exosome isolation module (for removing EV sub-parts). Kang et al. [105] carried out an efficient and satisfactory separation of exosomes from plasma, based on the adjoining effect of phosphatidylserine-specific proteins present on their surfaces associated with cancer. In another study, Ye et al. [100] used a microfluidic platform with protein chips in order to facilitate the separation and detection of exosomes [106,107]. Zeng et al. [108] utilized the microfluidic chip technique, combining it with a self-assembled three-dimensional herpet-shaped nanoplatform to capture and separate exosomes sourced from tumor cells, improving the separation efficiency of the process by several fold. Ding et al. [109] prepared a novel magnetic nanowaxberry (MNWB) microfluidic chip (EXoSIC) biosensor to efficiently isolate exosomes. The design of this chip is such that an irregular serpentine channel of this chip increased the chaotic mixing of exosomes containing fluid, hence improving exosomal capture efficiency. In comparison to the conventional spherical magnetic nanomaterials, MNWB not only shows enhanced capture efficiency but also has size exclusion impact, thereby improving the exosome purity. This technique showed 24 times higher exosomal yield and higher specificity than traditional centrifugation methods [109]. Summarily, microfluidic separation is a microscale breakneck separation technique, showing a wide application potential in an efficient recovery and higher sensitivity in performance, requiring lower sample volume and briefer treatment time [110].

### 3.6. Charge-Based Isolation

A few other techniques are being used efficiently to isolate exosomes, like ion (anion) exchange chromatography and electrophoresis. These techniques have popularly arisen as exosomes bear negative surface charges (due to membrane molecules like phosphatidylserine) [84]. In electrophoresis, exosomes carrying charges can be separated when placed under an electric field [111]. In the anion exchange chromatography (AIEX) technique, negative charges on the membrane interact with their positive counter-ion charges, eluting out EVs by providing additional buffers with high salt concentration and adjusting the ionic strength of the mobile phase [89,112]. A study based on this approach was carried out by Kim et al. [113] utilizing a centrifugal technique followed by performing an AEIX to separate exosomes generated by mesenchymal stem cells. Further, a comparative study was performed by Heath et al. [114] to compare the isolation efficiency of exosomes derived from HEK293T cells through AEIX, tangential flow filtration, and ultracentrifugation. It was found that the methods AIEX and ultracentrifugation provided almost equal yield and purity of exosomes. Moreover, AIEX was reported to be a single-step method that could be employed for scale-up studies having wide-scale clinical applications. Though these charge-based methods protect the integrity of sample compounds (exosomes), sometimes it is not suitable for certain biological samples, like blood bearing multiple charges [115].

Though there are various methods being employed for the isolation of exosomes, none of them until now have been completely successful [61]. Each isolation technique bears one downside or another. The major issue revolves around the compromised purity and physicochemical properties of exosomes, affected by different isolation techniques. To attain large-scale production of exosomes for clinical purposes remains another major challenge to be addressed [89]. Table 1 briefly covers the upsides and downsides of all the available major exosome isolation techniques as discussed in the previous sections.

## 4. Detection Methods for Exosomes

As exosomes present a huge number of significant applications in health science and medicine, identifying and detecting them appropriately becomes quite necessary in clinical research. Various detection techniques take the aid of recognition biomolecules such as nucleic acids, proteins, and lipids [118]. Nine and Llorente [119] and Rajput et al. [120] enlist various lipids, protein molecules, and nucleic acids associated with the biogenesis and release of exosomes, acting as a biomarker leading to their easy detection. Some of the commonly used methods for the detection of exosomes include optical, electrochemical assays [121], immunoreaction assays [122], aptamer-based detection [123], fluorescence [124], surface plasma resonance (SPR) [125], Surface-Enhanced Raman Scattering (SERS) [125], chromatography [126], and microfluidic detection methods [127]. One or more detection methods have also been combined to make the overall process more efficient.

### 4.1. Nucleic Acid-Based Detection of Exosomes

Nucleic acids reflect vital genetic information about the origin, function, physiological changes, and fate of existing exosomes, in the form of RNA, DNA, and microRNA [128]. These nucleic acid substances are free to transmit and convey information between the cells of the body, usually allied with disorders (particularly in the detection of tumors). This makes it feasible for RNA-based cancer drug therapy applications [129]. Typically, electrochemical assays and next-generation sequencing are used to detect nucleic acids. As the genetic materials (DNA/RNA) are predominantly allocated inside the exosome, they remain surrounded by the lipid bilayer, building a challenge of nucleic acid recognition during the diagnostic procedures. To overcome this, nucleic acids are first released, followed by identification. In a study, Zhang et al. [130] developed a method involving an adaptor magnetic bead as a bioconjugate, which induced the release of several mitochondrial DNAs (mDNAs) from LNCaP cells. The released mDNAs hybridized with the probe DNAs immobilized on a gold electrode. These mDNAs, detected via electrochemical signals, indicated the presence of tumor-associated exosomes.

In another study, Tan et al. [131] constructed an aptamer as electrochemiluminescence (ECL) for detecting cancerous exosomes from the hepatocyte cells, which relied on analyzing DNA nanostructure and nano-tetrahedron. The highest LOD of the used aptasensor system was determined to be 3.96 × 10^5^/mL [131]. In another study, Sun et al. [132] explored next-generation sequencing as also used for the detection of nucleic acids. Bovine milk was investigated on the basis of miRNA expression profile via next-generation sequencing. An upregulation in the level of exosomal miRNA indicated bacterial infection, highlighting the scope for early detection of bacterial infection in the mammary gland.

### 4.2. Protein-Based Detection of Exosomes

The literature review carried out so far suggests the presence of specific proteins on the surface of exosomes (also discussed in Section 2), providing their distinction from other vesicles and making them potent for use as markers in diagnostics [133]. Either a single type of protein can be utilized causing specific capture and detection of exosomes, or multiple distinct proteins can also be exploited for analyses of exosomes. Of the many proteins present on the exosome’s surface, tetraspanins are found in abundance, causing them to be absolute biomarkers for the quantitative analysis of exosomes. Similarly, exosomal protein CD24 and urinary exosomes with biomarkers (like fetuin-A and aquaporin-1) are associated with faster detection of autoimmune diseases and kidney injuries, respectively [134,135]. The proteins are commonly identified using immunoreaction, aptamers, and surface plasmon resonance-based methods.

#### 4.2.1. Aptamer-Based Detection

The word “Aptamers” has been taken from the Latin word *aptus*, which means “to fit”. They are synthetic ligands, emerging as an alternative to biorecognition elements, and are selected via the systematic evolutionary process of exponential enrichment of ligands (SELEX). Aptamers bear major properties of oligonucleotides or peptides with spatial conformational diversity. Proteins possess high specificity and precision while binding with aptamers, owing to a complementary shape and superposition, aided by intermolecular interactions, i.e., hydrogen and electrostatic bonds [7]. While comparing peptides (antibodies), nucleic acid aptamers possess a few added advantages, including their simple chemical modification, cheaper, better long-stage stability, and easy preparation. To date, several studies have reported the use of aptamers and a few other analytical techniques in conjugation, for instance, click-chemistry [136], colorimetric analysis [137], surface plasma resonance (SPR) [106], luminescent resonant energy transfer (LRET) [15], and giant magnetoresistance biosensors. Furthermore, some studies also report the use of combining antibodies (the immune proteins) with aptamers to avail an efficient and reliable quantification and detection of exosomes [138].

The approach of recognizing exosomes with surface-specific protein aptamers conjugated with fluorescence detection becomes an efficient practice and various studies support this technique through their research applications [139]. One such report was presented by Sun et al. [140], who built a thermophoretic aptasensor using seven fluorescent aptamers, which was dependent on the labelling of proteins present on the exosome’s surface. This approach was found to be efficient, highly sensitive, and cost-effective for detecting exosomes from a sample of prostate cancer [140]. Zhang et al. [141] used a fluorescence polarization approach, employing aptamers and the exosome’s membrane proteins (playing a key role) for the effective quantification of exosomes from human plasma, with a limit of quantification (LOQ) lying in the range of 5 × 10^5^ to 5 × 10^8^ particles/mL. Following a colorimetric analysis approach, Tan et al. [142] achieved a satisfactory level of exosome detection by allowing specific binding between the surface proteins (on the exosome’s surface) and the aptamers, leading to a visual color change, which was detected with the naked eye after a few minutes.

**Figure 9:**
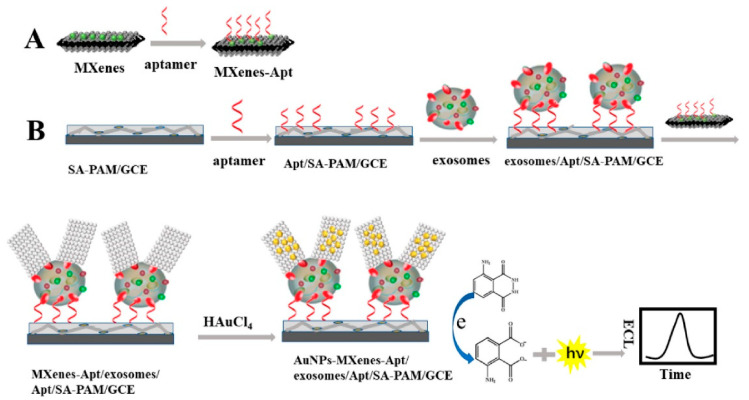
Schematic diagram representing exosome detection using ECL biosensor-based in situ formation of AuNPs with Ti_3_C_2_ MXenes hybrid. (**A**) Shows the binding of aptamer (Apt) on MXenes hybrid surface. (**B**) The SA-PAM layer provides carboxyl groups for the immobilization of CD63 Apt molecules, which enables an efficient capturing of exosome molecules, followed by their detection using ECL biosensor. After the exosomes being captured, the exosomes/Apt/SA-PAM/GCE surfaces were incubated with the MXenes-Apt solution based on high specific binding between Apt and CD63 protein on exosomes surface. At last, the modified surfaces were immersed in HAuCl_4_ solution (2 mg/mL) forming a AuNPs-MXenes-Apt/exosomes/Apt/SA-PAM/GCE complex. The resulting ECL signal was recorded in luminol solution. [SA-PAM/GCE: Sodium alginate-poly(acrylamide)/glassy carbon electrode; MXenes: 2D inorganic compounds consisting of thin layers of transition metals]. Adapted with permission from Zhang et al., 2020 [123]. Copyright 2020 American Chemical Society.

In line with this, Zhang et al. [123] used CD63 aptamer and modified AuNP (providing high electrocatalytic activity) with Ti_3_C_2_ to build a super perceptive ECL sensor for recognizing exosomes from cell lines (HeLa cells). The developed ECL biosensor was found to be a feasible, reliable, and highly sensitive detection system for clinical applications with an LOD value of 3 × 10^4^ particles/mL. Figure 9 represents the recognition process of exosomes using an ECL biosensor [123]. Moreover, Fang et al. [143] developed a dual-mode biosensor with both photothermal and ECL signalling. The system caused a reliable detection of exosomes as a result of EpCAM proteins (on the exosome’s surface) binding with the aptamers from the serum samples, giving the system an LOD value of 3.7 × 10^7^ particles/mL [143].

#### 4.2.2. Immunoreaction-Based Detection

The technique of using the immune response detected through the antigens and antibodies for recognizing proteins has been growing rapidly and is widely used for analyzing the diversity of exosomes [144]. Antibodies can be immobilized on surfaces via different linker molecules, such as glutaraldehyde, 1-ethyl-3-(3-dimethylaminopropyl) carbodiimide (EDC)/N-hydroxysuccinimide (NHS), and self-assembled monolayers (SAMs) [145,146]. In a study performed by Yuand et al. [122], an antibody-modified reduced graphene oxide was used to recognize the CD63 protein allocated on exosomes. The authors developed a field-effect transistor biosensor for the quantitative analysis of exosomes, showing a remarkable specificity and a low detection limit of 33 particles/μL. Moura et al. [147] used modified magnetic particles with bound CD81 antibodies and labelled exosomes with CD24 as enzyme-linked secondary antibodies, causing the immunomagnetic separation of exosomes through optical reading using a standard microplate reader. Immune assays can be combined with other detection techniques. In a study, Wang et al. [148] integrated immune assays with a surface acoustic wave (SAW) sensor. Gold nanoparticles (Au-NPs) were used in the SAW sensor. A carboxyl group was created between gold and sulfur via self-assembly of thioglycolic acid, leading to a better sensitivity for the detection of exosomes from the blood sample of cancer patients. For the further amplification of the detection signal, anti-CD63 was attached to the chip, and the corresponding EpCAM was taken as secondary antibodies to ensure specific rooting of exosomes with an LOD value of 1.1 × 10^3^ particles/mL.

#### 4.2.3. Surface Plasmon Resonance Imaging (SPRi)-Based Detection

**Figure 10:**
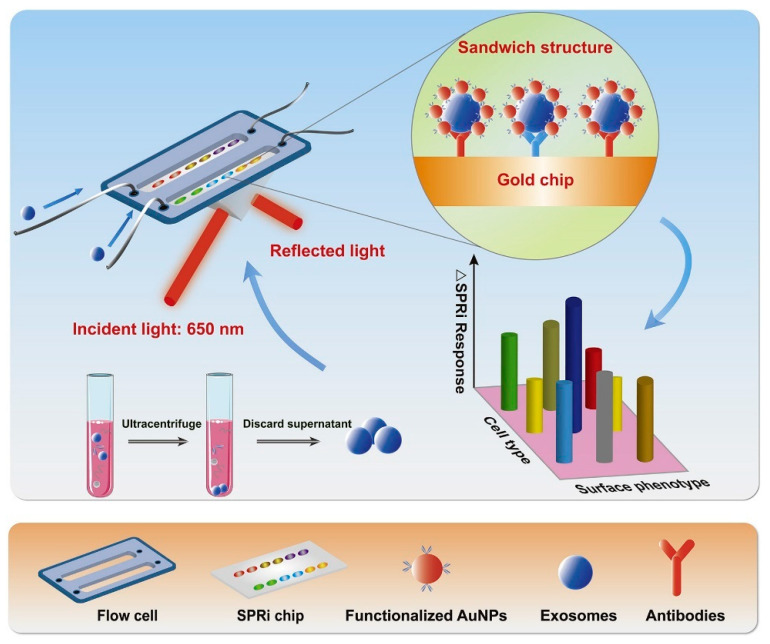
Working principle of SPRi biosensor for the detection of exosomes derived from NSCLC cells. AuNPs enhanced the signals. Adapted with permission from Fan et al., 2020 [149]. Copyright 2023 Elsevier.

The SPRi technique provides a real-time and label-free method for detecting exosomes. In this method, antibodies are attached to the sensor chips. Fan et al. [149] reported the use of SPRi biosensors bearing multiple recognition sites, each with diversified biological affinity of antibodies for multiple identifications of exosomes with higher sensitivity. The sample was taken from non-small cell lung cancer (NSCLC) cells. The performance of the SPRi biosensor system was enhanced with AuNPs. Figure 10 shows the working principle of the AuNP-enhanced SPRi biosensor for multiplex detection of exosomes derived from NSCLC cells [149]. The figure explains the exosome isolation using ultracentrifugation, followed by their detection using SPRi. Antibodies (anti-CD63, anti-EGFR, and anti-EpCAM) were attached to the sensor chip, and the supernatant containing exosomes flowed over it. Au-NPs functionalized with antibodies were also passed to amplify the signals. Different subtypes of exosomes having their corresponding conjugate antibodies on the SPRi chips were captured and detected. The LOD value was found to be 10^7^ particles/mL [149]. In another study, Picciolini et al. [150] used the SPRi method to provide a direct read-out of central nervous system status by detecting multiple neurogenic (brain-derived) exosomes directly from the blood plasma samples. This method showed an LOD value of about 1 μg/mL [150].

### 4.3. Lipid-Based Detection of Exosomes

As the outermost part of exosomes is composed of a lipid bilayer providing them inclusion stability and rich in components like cholesterol, phospholipids, and polyglycerol, a few approaches have been developed to target these lipid components to cause the specific detection of exosomes using the aptamer-based approach. This has caused a significant reduction in interference signals and ensured high specificity and sensitivity for exosome detection. Following the common recognition and detection of exosomes, lipids sometimes also adjoin to the surface protein present on the exosomes, known as double marker recognition. For instance, cholesterol is used as a probe for a target binding with aptamers or surface proteins like CD63, sometimes conjugated with a magnetic separation technique, enhancing the sensitivity of the method by several times [151].

**Figure 11:**
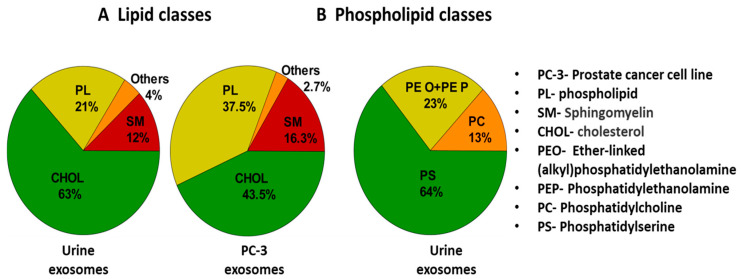
Pie chart showing the types of lipid molecules (**A**) and phospholipids (**B**) associated with exosomes along with their percentage abundance in urine sample. For comparison, exosomes from PC-3 cell line have been shown. Adapted and recreated with permission from Skotland et al., 2017 [152]. Copyright 2023 Elsevier.

In a study by Skotland et al. [152], various lipid molecules (present in exosomes) were detected and isolated from urine samples of patients suffering from prostate cancer. Further, using methods like mass spectrometry (MS) and lipidomics, quantification of different lipids was carried out. Figure 11 shows a pie chart comparing the types of lipid molecules (A) and phospholipids (B) associated with exosomes in a urine sample and prostate cancer cell line (PC-3) [152]. Phospholipid contents were higher in PC-3 cells. This indicated the diagnostic significance of exosomal lipids.

In one of the studies carried out by Zhang et al. [153], a cholesterol-modified DNA probe was developed to bind with CD63 aptamer, aided by magnetic bead marker separation to capture exosomes. The method was found to be easy and simple to perform. For better sensitivity and magnification of signals, hybrid chain reaction (HCR) of alkaline phosphatase was performed to analyze exosomes quantitatively through visual identification or via UV-vis spectrophotometer. The LOD for the developed detection technique was found to be as low as 1.6 × 10^2^ particles/mL, proving it to be an efficient and reliable exosomal detection and quantification system [153].

### 4.4. Label-Free Exosome Imaging Methods

**Figure 12:**
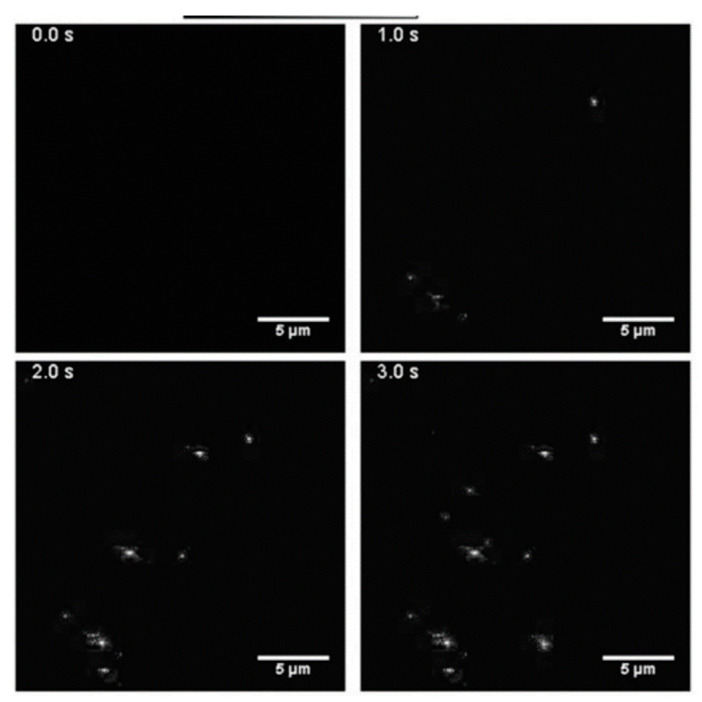
Detection of exosomes using iPM-based method. The bright spots represent the adsorption of exosomes onto positively charged surface, modified with gold. Scale bar: 5 µm. Adapted from Yang et al., 2018 [159].

Exosomes can be detected, and their size can be analyzed based on an optical imaging approach. It is performed in a label-free manner with the help of interferometric plasmonic microscopy (iPM) or plasmonic scattering microscopy (PSM) [154,155]. Though labeling methods like fluorescence microscopy, it possesses high sensitivity and is a powerful technique, but in some cases, the labelling of target molecules becomes challenging and may lead to some dubious changes [156]. Therefore, there is a high demand for label-free methods with improved sensitivity and a wide visual range to clearly envision nanoparticles associated with various biological events [157]. PSM has proven to be an incredible tool in analyzing exosomes, as it can easily distinguish the samples (with a size range of 200 nm), rejecting noise and disturbances from the out-of-focus medium. iPM is constructed in the Kreschmenn configuration and is akin to a surface plasmon resonance microscope (SPRM), with certain added advantages like higher sensitivity, lack of distortion, and better spatial resolution [158]. In a study by Yang et al. [159], the surface was chemically modified using gold, and a real-time adsorption study of exosomes was conducted on the modified surface. Figure 12 represents the snapshots of distinguishable bright spots of exosomes adsorbed over gold-modified surfaces through iPM images, bearing positive charges (as exosomal particles carry negative surface charges). Further, the image intensity and size distribution of exosomal particles were determined. The method also allowed quantitative measurement of the membrane fusion activity between exosomes and liposomes as well as the monitoring of the driving interaction between exosomes and antibodies. In another study by Zhang et al. [154], PSM was used to determine the size distribution of exosome particles and quantify their binding kinetics without labels by flowing two EV solutions, providing high-resolution images. The size (mean diameter) of exosomal particles was found to be nearly 115.8 nm for the 5 × 10^7^/mL EV solution and 116.6 nm for the 5 × 10^10^/mL EV solution [154].

### 4.5. Nanoplatforms and Nano-Biosensors for the Detection of Exosomes

Nanoparticles provide a large specific surface area for the efficient binding of exosomes. Nowadays, more emphasis is given to magnetic nanoplatforms and nano-biosensors to detect and capture exosomes, as they can be easily separated under magnetic fields. Furthermore, these nanomaterials can be conjugated to target specific ligands in order to impart target specificity. However, nanoparticles may not possess surface functional groups to which the other entities (i.e., antibodies, aptamer) can be conjugated. For this purpose, surface functionalization/modification of nanoparticles is performed [160,161,162,163,164,165]. Different self-assembled monolayers (SAMs) are formed on the surfaces, which provides functional groups and also alters surface energy [116,166]. Silane or thoil reagents are used for this purpose. For example, 3-aminopropyltriethoxysilane (APTES) and octadecyltriethoxysilane (OTES) provide -NH_2_ and -COOH groups, respectively, on surfaces [166,167,168]. Ligands can be directly attached to surfaces with SAMs. However, linker molecules like glutaraldehyde or 1-ethyl-3-(3-dimethylaminopropyl) carbodiimide (EDC)/*N*-hydroxysuccinimide (NHS) are exploited to conjugate target-specific entities for the efficient detection of exosomes.

**Figure 13:**
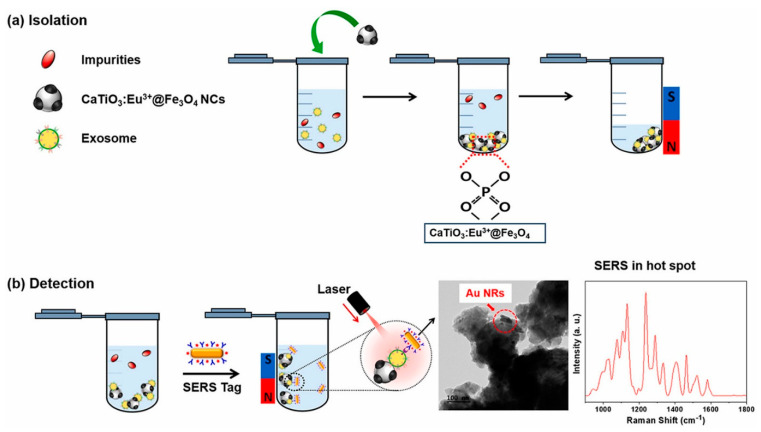
(**a**) Schematic of isolation and magnetic separation of exosomes using CaTiO_3_:Eu^3+^@Fe_3_O_4_ nanocomposite. (**b**) SERS−based immune−assay for the detection of exosomes based on CD8. Adapted from Back et al., 2023 [169].

The designed nanoplatforms exploit the biomacromolecules present in exosomes for detection. Protein and lipid-based detections are commonly integrated. In a study, Back et al. [169] developed a simple, rapid, and efficient strategy for the isolation and detection of exosomes via a multifunctional nanocomposite of CaTiO_3_:Eu^3+^@Fe_3_O_4_. The nanocomposite was synthesized using a high-energy ball milling process. Exosomes were captured by the binding of the hydrophilic phosphate head group of exosomal phospholipids to CaTiO_3_:Eu^3+^@Fe_3_O_4_. Further, a SERS-based immune assay was used to detect the target antigen (CD81). The schematic of the exosome capture and magnetic separation is shown in Figure 13a and the SERS-based detection process is shown in Figure 13b. This study proposed a multifunctional nanocomposite for the capture and rapid detection of exosomes [169]. In another study, Wang et al. [170] synthesized a guanidine-functionalized (GF)-covalent organic framework (COF) nanocomposite with a layer-by-layer approach to capture exosomes and phosphopeptides. Fe_3_O_4_@COF provided a large number of binding sites for AuNPs, which were functionalized with amine groups using polyethyleneimine (PEI). The composite was further grafted with guanidine and designated as Fe_3_O_4_@COF@Au@PEI-GF. The exosomes were captured from a human serum sample, and their size was found to be in the range of 30–150 nm, as analyzed using TEM. Nanoparticle tracking analysis (NTA) [5] was used to track the real-time dynamic Brownian motion of the nanoparticles in order to estimate the concentration of the captured exosomes. The captured exosome concentration was found to be ~1.2 × 10^9^ particles/mL. The exosomal capture can be described by the interactions between the guanidyl groups of Fe_3_O_4_@COF@Au@PEI-GF and the phospholipid layer of exosomes [170].

Jang et al. [171] explored the protein-based detection method. Transferrin-combined magnetic nanoparticles (MTNs) were used to isolate brain blood-derived exosomes in neurological diseases. Silica-coated Fe_3_O_4_ (Fe_3_O_4_@SiO_2_-NH_2_) nanoparticles were synthesized and further conjugated to transferrin. Transferrin here acted like a ligand as it can bind the transferrin receptor present on the surface of exosomes. The study envisioned the potential of MTNs to not only isolate blood-derived exosomes but also for other exosome-related theranostic applications [171]. In a similar study by Farsani et al. [172], silica shell-coated magnetic nanoparticles (MSNPs) with a diameter of ~140 nm were used to isolate exosomes. These MSNPs were conjugated to Anti-CD9 antibody and to carboxylated (CMSNPs) or aminated MSNPs (AMSNPs). The results showed >90% recovery of exosomes using both AntiCD9-CMSNPs and AntiCD9-AMSNPs [172]. In another study, Singh et al. [173] designed a co-capture-based approach for the enrichment and detection of lung cancer-derived exosomes. It used a sandwich technique, which involved a CD151 antibody coupled to magnetic nanoparticles for immune-magnetic selection of target exosomes and CD81 as a secondary antibody conjugated with HRP for the amplification of the signal. This approach isolated 10^5^–10^8^ exosomes/mL with an LOD value of 6 × 10^4^ exosomes/mL [173]. The researchers then studied plasma samples of 18 healthy people and lung cancer patients. This nanoassembly could differentiate between healthy individuals and lung cancer patients by quantifying CD151+/CD81+ lung-derived exosomes [173].

**Figure 14:**
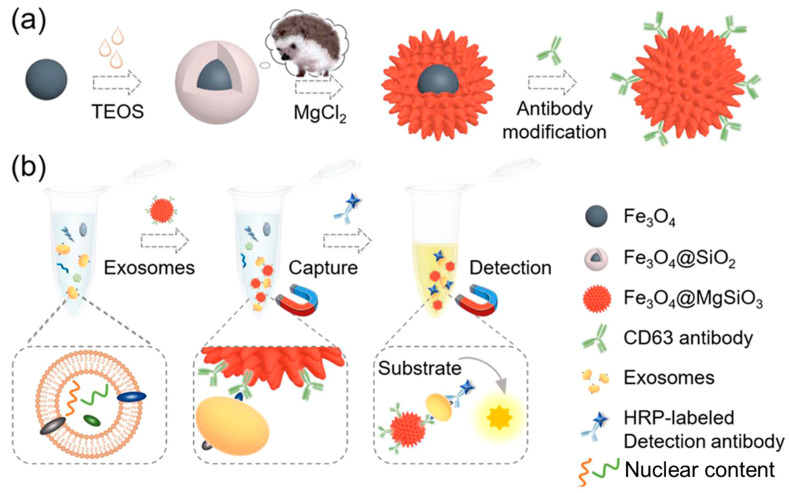
(**a**) Schematic of nanoplatform preparation and modification. (**b**) Illustration of exosome isolation and detection by the prepared Fe_3_O_4_@MgSiO_3_@CD63 nanoplatform. Adapted from Yang et al., 2021 [174].

Inspired by the hedgehog burr-like structure, Yang et al. [174] synthesized magnetic nanoparticles having their surface covered with nano-needles to capture and detect exosomes. Magnetite (Fe_3_O_4_) nanoparticles were synthesized by a hydrothermal method, and then surface coating was carried out using tetraethylorthosilicate (TEOS) to obtain Fe_3_O_4_@SiO_2_. The obtained nanoformulation was heated with magnesium salt in a hydrothermal chamber to obtain Fe_3_O_4_@MgSiO_3_. Further, a CD63 antibody was conjugated to Fe_3_O_4_@MgSiO_3_ through co-valent modification in an EDC/NHS reaction solution. The synthesis and surface functionalization approaches are shown in Figure 14a. The clinical samples were collected from patients with liver cancer and healthy individuals. It was found that with an increasing concentration of CD63 antibody (from 0.001 to 10 µg/mL), exosomal capture was increased. HepG2-derived exosomes can thus be used as biomarkers for disease diagnosis owing to the excellent exosome capture efficiency of the designed nanoplatform. The schematic of exosome detection and capture using Fe_3_O_4_@MgSiO_3_@CD63 is shown in Figure 14b.

**Figure 15:**
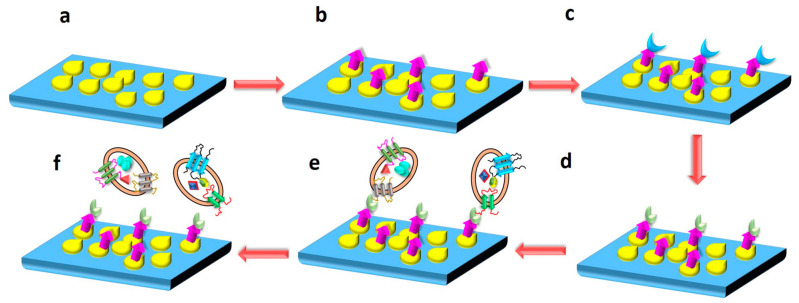
The nanoplatform presented here consists of a silicon wafer shown in blue, onto which AuNPs are coated (yellow moieties). Further, PEG conjugation is done, represented by purple color. EDC/NHS reaction was carried out to activate PEG, as shown by green color. Exosomes from patient serum sample were immobilized onto the nanocomposite, as shown by oval shaped moieties. The schematic of the process depicts: (**a**) AuNP-coated Si- wafer. (**b**) PEG conjugation on AuNP-coated Si wafer. (**c**) EDC/NHS reaction for the activation of PEG. (**d**) Anti-CD63 antibody conjugation to Si wafer via PEG. (**e**) Immobilization of exosomes on the surface of Si wafer after incubation with patient serum sample. (**f**) Eluting exosomes from the surface of Si wafer. Adapted from Pammi et al., 2023 [175].

In another study, a silicon (Si) wafer coated with AuNPs and functionalized with polyethylene glycol (PEG) was used to isolate exosomes from a serum sample. PEG was used to further conjugate anti-CD63 antibody on the surface of the nanocomposite via EDC/NHS reaction. The Si wafer was then incubated with the serum to immobilize exosomes on its surface by binding anti-CD63. Western blotting was carried out, and the presence of heat shock protein (HSP70) and calnexin confirmed exosome elution/isolation. The elaborated schematic representation of the fabrication of Si wafer surface for immobilizing exosomes is depicted in Figure 15. The advantage of the proposed technique could be the reusability of the Si wafer, and it also allows for the isolation of other sub-ranges of exosomes by altering the size of NPs [175]. Microfluidic technology is a powerful tool to design and develop lab-on-chip biosensors for the efficient detection and isolation of exosomes. In recent years, there have been attempts to design such biosensors [127]. In a study by Ding et al. [109], as discussed in Section 3.5 above, a novel magnetic nanowaxberry (MNWB) microfluidic chip biosensor was designed to efficiently isolate exosomes with an LOD value of 2.4 × 10^7^ particles/mL [109].

## 5. Applications of Exosomes

**Figure 16:**
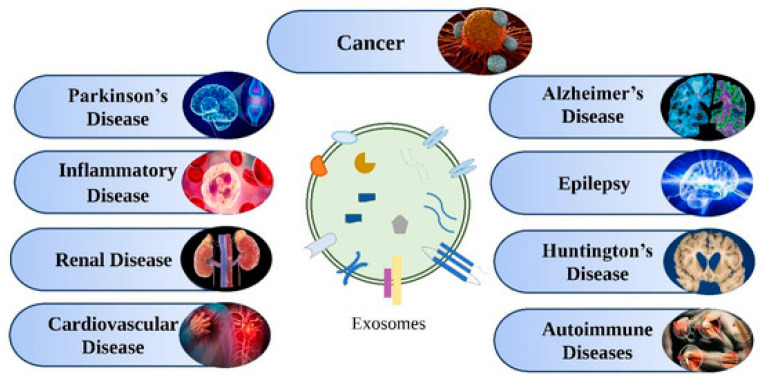
Various diseases for which exosomes act as therapeutic agents and biomarkers. Adapted from Rajput et al., 2022 [120].

Exosomes are present in many bodily fluids, such as serum, saliva, urine, cerebrospinal fluid, and breast milk, and are also found in various cells, such as immune cells (B cells and T cells), dendritic cells, mast cells, and platelets. Several reports on exosomes have revealed their potential as biomarkers for the detection of several diseases, drug delivery, and therapeutic agents. The molecular contents of exosomes, such as proteins, lipids, and nucleic acids, remain in a stable state and thus can be used as therapeutic agents as well as biomarkers for diseases, such as neurodegenerative disorders, viral infections, diabetes, autoimmune syndromes, and cancer, as shown in Figure 16. A few selected applications of exosomes for diagnostics and therapeutics are discussed in the following sections.

### 5.1. Exosomes for Early Detection of Diseases

#### 5.1.1. Neurodegenerative Diseases

##### Parkinson’s Disease

Parkinson’s disease (PD), being a neurodegenerative disorder, is associated with synaptic dysregulation and neuronal death. Conversely, there is no effective diagnostic biomarker available for the early detection of PD. Recently, Bhattacharyya et al. [176] reported the presence of brain-enriched microRNAs (miRNAs) in circulating exosomes. The researchers isolated exosomes from the blood sample of PD patients and found that miR-128 was found circulating in synaptic vesicles of PD patients. The study showed that over-expression of miR-128 can control mitochondrial superoxide production and can also prevent downregulation of pre-synaptic terminal protein (Synaptophysin) in PD pathogenesis. Altering the expression of exosomal miR-128 can thus play an essential role in the pathogenesis and detection of PD [176]. There are a few other reports on a range of miRNAs present in circulating exosomes in PD patients. These miRNAs could be explored as biomarkers for the early detection of PD [177,178,179,180]. On the other hand, Tomlinson et al. [181] explored the immuno-typing of the circulating exosomes. It was found that among 1033 proteins identified in the sera of PD patients, 23 exosomal proteins are abundantly present in PD. The researchers studied the changes in distinct proteins, while there was no significant change in microvesicles. This suggested that there exists a subpopulation of exosomal protein-enriched microvesicles in PD. This study gave in-depth insight into the proteomic analysis or profiling of some specific exosomal subpopulations that could be useful biomarkers for neurodegenerative diseases [181].

##### Alzheimer’s Disease

Yang et al. [15] reported the differential expression and cut-off values for miRNAs (miR-135a, miR-193b, and miR-384) for the diagnosis of Alzheimer’s disease (AD). The expressions of miR-135a and miR-384 were upregulated in the serum of AD patients, while the expression of miR-193b was downregulated [15]. In addition to nucleic acids, AD is characterized by the aggregation of Aβ peptide and tau protein [182,183,184]. In a study, blood samples from healthy people and AD patients were collected to capture exosomes [12]. The study revealed that the expression of AD-related proteins (Aβ1–42 and P-S96-tau) was found to be higher in the samples of AD patients as compared to healthy people. Fe_3_O_4_@Au@aptamer was used to detect the exosomes possessing CD63 markers on their surface [12]. These findings indicated that the differences observed in the exosome analysis of healthy people and AD patients could thus act as biomarkers for the early detection of AD [12].

##### Amyotrophic Lateral Sclerosis

Exosomal miRNAs have been studied by various research groups to reveal their potential as biomarkers for another neurodegenerative disease, amyotrophic lateral sclerosis (ALS) [185,186]. Lo et al. [187] observed the alternation in the expression levels of extracellular vesicles or exosome-derived miRNAs in patients suffering from ALS as compared to healthy individuals. The results show an increased level of miR-342-3p and a decrease in the levels of miR-1254 in three tissues of ALS patients. Additionally, an overlap was observed among miR-587, miR-298, miR-4443, and miR-450-2-3p across two tissues. This dysregulation associated with neurodegeneration causing ALS could pave the way for the identification of potential biomarkers of the disease [187]. Similarly, Kim et al. [188] studied small RNA sequencing of 18 ALS patients and 15 healthy individuals. It was found that five of the miRNAs are differentially expressed in the patients of ALS as compared to the healthy individuals. Furthermore, the results showed that miR-23c was upregulated, whereas miR-192-5p was downregulated in ALS patients. Bioinformatic analysis revealed that these miRNAs interact with different sets of genes which are involved in pathogenies in ALS. So, these two miRNAs can serve as potential biomarkers for detecting ALS [188]. In another study by Saucier et al. [189], it was found that miR-15a-5p could serve as a biomarker for the diagnosis of ALS, as miR-193a-5p is linked to the progression of ALS. Banack et al. [190] stated that miRNA sequences from neural-enriched extracellular vesicles of ALS patients may give valuable insights into the mechanism of neurodegeneration and can help in the early detection of ALS.

#### 5.1.2. Cancer

For the detection of cancers, biopsy is commonly used, which is invasive and not a patient-friendly approach. The post-surgery lesions and risk of infections are major concerns. Moreover, accessing the suspected region can also be challenging. For that matter, researchers are extensively working towards novel approaches for early detection [191,192]. In this regard, exosomes possess great potential for diagnosis by acting as biomarkers. Exosomes present in tumors contain tumor-specific proteins that are involved in tumorigenesis. Thus, tumor-derived exosome isolation and detection can be a promising approach for early detection of cancers.

##### Prostate Cancer

Prostate-specific membrane antigen (PSMA) can be used as a biomarker for prostate cancer diagnosis. Li et al. [193] synthesized a dual-functionality nanocomposite of Fe_3_O_4_@SiO_2_@TiO_2_ with reversible conjugation and on–off signal response for exosome isolation. TiO_2_ binds reversibly with the phosphate groups of the lipid bilayer of exosomes. The exosomes were isolated from the serum sample of healthy donors and prostate cancer patients. NTA analysis estimated the concentration of exosomes to be 3.21 × 10^10^ particles/mL with an LOD value of 5 × 10^5^ particles/mL. The study showed that PSMA was over-expressed in patients with prostate cancer, indicating the exosomes in serum as a useful biomarker for the diagnosis [193]. In another study, an anti-prostate-specific antigen (tetraspanin) was immobilized onto Ag/Fe_3_O_4_/graphene surface to isolate prostate cancer-specific exosomes [194]. The antibody was conjugated to a dye, and depending on the exosomal concentration, the fluorescence intensity varied. This system can be exploited as a potential platform for the biosensing of prostate cancer.

##### Breast Cancer

Breast cancer is one of the major malignancies leading to deaths in women. It is very important to detect it in the early stage. Li et al. [195] isolated exosomes and detected their breast cancer-specific markers by SERS. The magnetic SERS nano-platform was conjugated with anti-CD9. The designed SERS platform was able to bind specifically to targeted exosomes and could distinguish exosomes isolated from different cancer cell lines (MCF-7 cells and MDA-MB-231) [195]. In another study, Qin et al. [196] designed an interesting dual-cycling nanoprobe for the effective detection of miR-21 in blood plasma samples of breast cancer patients [196].

The simultaneous detection of different types of cancers is also possible using exosomes. In a study, Shin et al. [197] collected plasma samples from 210 healthy people and 543 cancer patients. AuNPs aggregated on APTES-functionalized glass surface array chip were prepared for SERS-based detection. Exosome markers such as CD9, CD63, CD81, and TSG101 were identified from cancer patients. The various biomarkers targeted using nanoplatform/nano-biosensor-conjugated aptamers/antibodies along with their capture efficiency have been listed in Table 2 for the early detection of diseases.

### 5.2. Therapeutic Potential of Exosomes

Stem cell-derived exosomes are very useful for pre-clinical and clinical studies for the treatment of heart strokes, myocardial infarction, and acute kidney failures. Mesenchymal stem cells (MSCs) are reported to secrete anti-inflammatory protein, namely TSG-6, which plays an important role in myocardial infarction [199]. In a study, a reduction in infarct size was observed in myocardial ischemic mice with the use of MSC-derived exosomes [200]. It has also been reported that MSC-derived exosomes help in increasing neurite branch numbers and neurite lengths in the case of the cerebral artery occlusion model of stroke [13]. Cardiac cell-derived exosomes also play an essential role in restoring cardiac functions in the case of myocardial infarction. These healing effects are mediated by the upregulation of several miRNAs, for instance, miR-21, miR-4454, and miR-125b [201].

Exosomes have been exploited for other chronic diseases like cancer and diabetes. Natural killer cell-derived exosomes could inhibit tumor progression by lysing tumor cells and delivering cytotoxic molecules. Chimeric antigen receptor-presenting cell (CAR-T cell)-derived exosomes are also reported to exert cytotoxic effects against cancer cells [202,203]. Exosomal miRNAs, such as miR-223, miR-29, miR-103, miR-107, and let-7, are reported to regulate diabetes via various molecular pathways, namely modulation of lipid and/or glucose metabolism, regulation of insulin secretion, liver gluconeogenesis, and autophagy [16,204].

Exosomes are also useful in wound healing and bone tissue engineering. MSC-derived exosomes are also exploited for wound healing. It was observed that human umbilical cord-derived MSC exosomes enhance skin cell proliferation, whereas apoptosis of skin cells is found to be inhibited by these exosomes in the case of rat burn models. Upon treatment of the burn wounds with MSC-derived exosomes, re-epithelization was found to be accelerated with an increased expression of PCNA, CK19, and collagen 1 [14]. MSC-derived exosomes are also reported to be useful in bone fracture healing, as they can promote osteogenic differentiation of bone marrow stem cells [205,206].

### 5.3. Exosomes as Drug Delivery Vehicles

**Figure 17:**
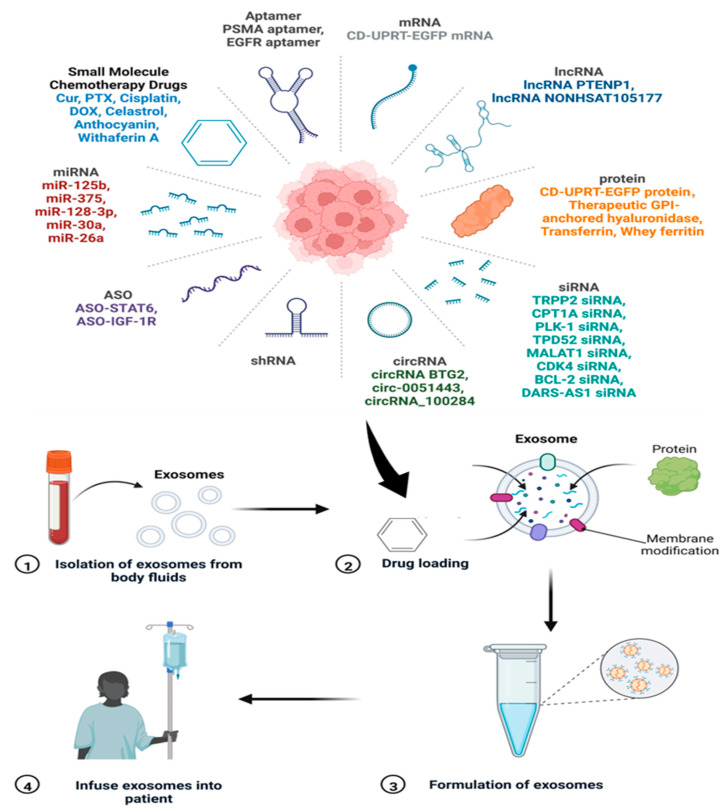
The process of drug loading onto the exosomes and their infusion in patients. The various drugs/cargoes which can be loaded on exosomes are also shown. Adapted and recreated from Zhang et al., 2022 [207].

There are various polymeric materials, liposomes, and nanomaterials that are being used as advanced drug delivery systems (DDSs). These DDSs are used to deliver antimicrobial, antiviral, and anticancer drugs to the target. However, the compatibility issues with the host cells remain challenging for using these materials as DDSs. In this direction, exosomes are emerging as novel nanoscale DDSs with the advantages of being biocompatible, target-specific, and sustainable. Exosomes can cross the blood–brain barrier (BBB) and mediate inter-cellular communication. Schematics of the drug loading, formulation, and delivery processes using exosomes are depicted in Figure 17. The figure also represents various cargo/drug molecules like small molecules, aptamer, RNAs, and proteins.

An overview of exosomes as DDSs for delivering biopharmaceuticals and their clinical application has been discussed by Butreddy et al. [208]. Kim et al. [209] summarized exosome application as DDSs for cancer therapy with a focus on delivering various types of cargo to the target. Interestingly, exosomes possess a tendency to accumulate in cancerous tissues more than normal cells, which makes them a preferable choice to be used as anticancer drug carriers [120]. Gomari et al. [210] showed that exosomes labelled with PKH67 are preferentially uptaken by breast cancer (HER2+) cells. The unlabelled exosomes, on the other hand, showed negligible binding to HER2+ cells. Moreover, the authors studied the effect of free doxorubicin (DOX) and DOX-loaded exosomes on BT-474 and MDA-MB231 cell lines. The results showed no significant difference in the toxicity of free and encapsulated DOX, while reducing the side effects in the case of the exosome-loaded drug [210].

The suitability of exosomes as DDSs for miRNA-based therapy was evaluated by Lorena et al. [211]. Bovine milk-derived exosomes were used to deliver extracellular miRNAs to the target. The exosomes were isolated from cow milk using a combined isolation methodology including ultracentrifugation and SEC. Afterwards, the isolated exosomes were loaded with hsa-miR148a-3p, an exogenous miRNA that is highly expressed in milk exosomes. The absorption of loaded miRNA by hepatic and intestinal cell lines (HepG2 and Caco-2) was evaluated to check the potential of possessed DDS for the delivery of miRNAs [211]. Interestingly, 20- and 30-fold increases in the concentration of miR-148a-3p were observed at 2 h in HepG2 and Caco-2 cells, respectively [211].

Furthermore, surface functionalization of exosomes can be carried out to make them highly target-specific. In a study by Tian et al. [212], (cRGDyK) peptide was conjugated to exosomes to target ischemic brain lesions. The engineered exosomes (cRGD-Exo) were further loaded with curcumin to suppress the inflammatory response and cellular apoptosis. The study provided a strategy for designing engineered exosomes to treat lesion regions in the ischemic brain [212]. Despite having ample literature on the therapeutic potential of exosomes, their practice in clinics is still limited. The clinical trials of exosome-based therapeutics are at the initial stages, as listed in Table 3. Major developments are in phase I of the clinical trial.

## 6. Conclusions and Future Outlook

Exosomes actively participate in cargo transportation and intercellular communication. Owing to their bio-physiological properties, they are considered the best biomarkers for several diseases, such as cancer, autoimmune syndromes, neurogenerative disorders, and diabetes. Moreover, exosomes can serve as drug delivery agents and possess therapeutic potential. Therefore, the analytical and isolation methods for exosomes have attracted a lot of research. The traditional methods for the isolation and detection of exosomes include centrifugation, immunoaffinity, size-based isolation, and cellular component-based identification. However, highly efficient, rapid, and technically developed methods are desirable. Despite the increasing interest of researchers in this field, a highly sensitive and reliable isolation method remains a challenge. In this context, extensive research is being carried out to develop and design multifunctional and highly efficient nanomaterials and nano-biosensors. These nanoplatforms and biosensors are designed to target over-expressed biomarkers on the surface of exosomes, for instance, CD63, CD9, and CD81, to name a few, and cellular components, such as proteins and exosomal miRNAs. Working protocols, limit of detection, and breakthroughs in designing highly efficient and multifunctional nanoplatforms and nano-biosensors hold promise for the efficient isolation of exosomes and early detection of diseases. Furthermore, a standard protocol needs to be established for exosomes isolated from different sources to prevent batch-to-batch variations and create reproducibility.

Exosomes isolated from diseases have the potential for clinical diagnosis and early detection of the disease by identifying target molecules. Biomacromolecules like nucleic acids, proteins, and lipids are being exploited for detection. Further, technical developments in traditional isolation and detection methods are being carried out. Nanomaterials possess the potential for fabricating highly sensitive, reliable, and robust biosensors for clinical application and commercialization. Novel approaches, such as microfluidics, are needed to develop next-generation biosensors or lab-on-chips. Furthermore, future research in this field should also focus on lipid profiling apart from proteomics and/or genomics of exosomes to discover novel exosomal biomarkers for the early detection of diseases, as lipids are readily available on the surface of the exosomes.

Interestingly, exosomes derived from various body cells hold therapeutic potential. MSCs and cardiac cell-derived exosomes are useful in the treatment of heart ailments. Various exosome-derived miRNAs are helpful in the treatment of bone fractures, skin burns, and liver-related dysfunctions. Tumor cells could also be lysed by exosomes, thereby inhibiting tumor progression. In-depth insight into the mechanism(s) is required to see how exosomal machinery is involved in treating various diseases. Moreover, there is still a major challenge to be addressed, i.e., cost-effective and reproducible large-scale production of exosomes. As exosomes represent promising future nano-medicine for the cure of various ailments, their production on a large scale is highly demanding.

## Figures and Tables

**Table 1:** The advantages and disadvantages of techniques being used for the isolation of exosomes.

Isolation Techniques	Advantages	Disadvantages	References
Ultracentrifugation	Cheaper Simple steps Multiple samples can be isolated in one go through differential centrifugation	Long treatment time Low recovery rate Low purity of extracted products Labor-intensive Additional steps for purity may be required Isolated product prone to degradation	[33]
Precipitation	Highly efficient Cost-effective Easy to perform Suitable for both high and low sample concentrations	Exosomes are usually isolated along with impurities Complicated cleaning steps Lengthy process	[77,116]
Immunoaffinity	High specificity Simple operation Devoid of chemical contamination	Elution steps for exosomes needed The activity of exosomes prone to salt concentration and pH Low yield Costly antibodies required	[79,117]
Ultrafiltration	Simple operation Efficient in removing impurities Fast procedure No special equipment required Fine portability	Time-demanding Expensive High chances of protein contamination Possibility of exosome loss by getting trapped in membrane filters	[89]
Size exclusion chromatography	Efficient in performance Maintains biological activity and integrity of the sample	High-quality chromatographic column required Low reusability Sample dilution Time-demanding	[88]
Microfluidic separation	Efficient recovery Very small sample volume Short treatment time (rapid) High-throughput analysis and automated capacity Real-time process control Exosome isolation and detection are possible simultaneously	High signal-to-noise ratio Low sample capacity Scaling up of process is challenging Expensive device development	[100,110]
Charge-based isolation	Maintains structural and functional integrity of the sample	Not often used for biological samples with multiple charges	[103]

**Table 2:** Various biomarkers targeted using nanoplatforms/nano-biosensors and their capture efficiency for the early detection of diseases. Here, NA refers to “Not available.”

Disease	Biomarker(s)	Nanoplatform Used	Antibody/Aptamer	Method of Detection	Isolated Exosome Concentration (Particles/mL)	Capture Efficiency (%)	Ref.
Parkinson’s disease	miR-128	NA	p-FoxO3a (Ser253)	NA	NA	NA	[176]
Parkinson’s disease	23 exosomal proteins	NA	Rabbit anti-flotillin, rabbit anti-Tsg101, rabbit anti-syntenin 1	Immuno-typing	NA	NA	[181]
Amyotrophic lateral sclerosis	miR-23c and miR-192-5p	NA	HRP-conjugated goat anti-mouse antibody	NA	NA	NA	[188]
Amyotrophic lateral sclerosis	miR-15a-5p and miR-193a-5p	NA	NA	NA	NA	NA	[189]
Amyotrophic lateral sclerosis	miR-342-3p and miR-1254	NA	anti-β-actin	NA	NA	NA	[187]
Alzheimer’s disease	Aβ1-42 and P-S96-tau	Fe_3_O_4_@Au@aptamer	Anti-CD63 antibody	NA	NA	NA	[12]
Alzheimer’s disease	miR-135a, miR-193b and miR-384	NA	NA	NA	NA	NA	[15]
Prostate cancer	Prostate specific membrane antigen (PSMA)	Fe_3_O_4_@SiO_2_@TiO_2_	Anti-PSMA, anti-CD9, and CD63	Fluorescence	3.21 × 10^10^	91.5%	[193]
Prostate cancer	Tetraspanin	Ag/IO/GRP	Dye-tetraspanin antibody	Magnetofluoro-immunosensing	NA	NA	[194]
Six different cancers	CD9, CD63, CD81 and TSG101	NA	NA	SERS profiling using AI	10^9^–10^10^	~90	[197]
Breast cancer	CD9	MNPs@PEI@MUA	Biotin	SERS	NA	~91	[195]
Breast cancer	PD-L1 and miR-21	NA	NA	NA	NA	NA	[196]
Colorectal cancer	CD63	Magnetic beads coated with carbon nanomaterial	Anti-CD63 antibody	Aptamagnetic-fluorescence sensing	1457	NA	[198]

**Table 3:** Current status of the clinical trials for use of exosomes in the treatment of various diseases.

Disease	Exosome	Mode of Administration	Administration Dosage and Duration	Clinical Trial Phase	Recruitment Status	Ref. @
Knee Osteoarthritis	MSC-derived exosomes	Intra-articular	3–5 × 10^11^ particles/dose	Phase I	Not yet recruiting	NCT05060107
Type I Diabetes Mellitus (T1DM)	Blood-derived exosomes	Intravenous	120–160 mg/dL	Phase I	Unknown	NCT02138331
Decompensated Liver Cirrhosis	Umbilical cord-derived MSC exosomes	Not specified	40 mg in three weeks	Phase II	Recruiting	NCT05871463
Skin Rejuvenation	MSC-derived exosomes	Intravenous	Not specified	Phase I/II	Recruiting	NCT05813379
Colon cancer	Curcumin-conjugated plant exosomes	Oral	3.6 g (gm) taken daily for 7 days	Phase I	Unknown	NCT01294072
Pancreatic Adenocarcinoma	MSC-derived Exosomes with KRAS G12D siRNA	Not specified	15–20 min on days 1, 4, and 10	Phase I	Recruiting	NCT01294072
Alzheimer’s Disease	Allogenic Adipose MSC- derived exosomes	Nasal	5–20 μg for 12 weeks	Phase I/II	Unknown	NCT04388982
Alzheimer’s Disease	Blood neuro-exosomal synaptic proteins	Not specified	Not specified	Not specified	Not yet recruiting	NCT05163626

@ ClinicalTrials.gov (accessed on 29 June 2023) [213].

## Data Availability

No new data were created.
